# Supplementary material for: Optimized feature gains explain and predict successes and failures of human selective listening
Source: bioRxiv. 2025 May 28:2025.05.28.656682. Preprint. [Version 1] doi: 10.1101/2025.05.28.656682 (PMC12154610; doi:10.1101/2025.05.28.656682)
Supplement: 1 [file NIHPP2025.05.28.656682V1-supplement-1.pdf]

bioRxiv preprint doi: https://doi.org/10.1101/2025.05.28.656682; this version posted May 28, 2025. The copyright holder for this preprint (which was not certified by peer review) is the author/funder, who has granted bioRxiv a license to display the preprint in perpetuity. It is made available under aCC-BY 4.0 International license.

Supplementary Figures and Tables for Griffith, Hess & McDermott

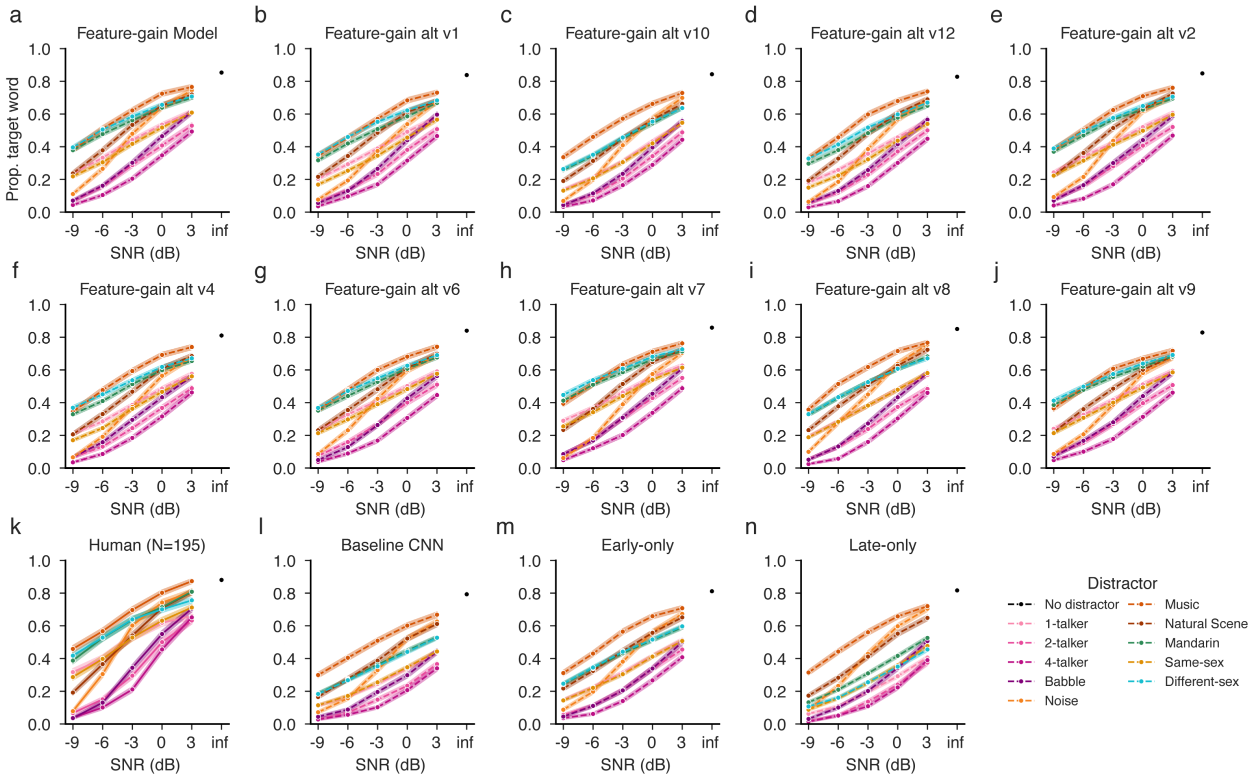

Supplementary Fig. 1 | Performance of all models on Experiment 1. Word recognition as a function of signal-to-noise ratio and distractor type for each model architecture. Panels a-j show results for each individual feature-gain model architecture (main figures show results averaged across these ten architectures, with different conditions plotted separately to elucidate particular effects of interest). Panel k shows results for human participants, replotted from Figure 2. Panels l-n show the baseline, early-only, and late-only architectures which each perform worse with speech-on-speech examples than both humans and the feature-gain models, particularly with 1-distractor (light-pink, gold, green, and blue lines).

bioRxiv preprint doi: https://doi.org/10.1101/2025.05.28.656682; this version posted May 28, 2025. The copyright holder for this preprint (which was not certified by peer review) is the author/funder, who has granted bioRxiv a license to display the preprint in perpetuity. It is made available under aCC-BY 4.0 International license.

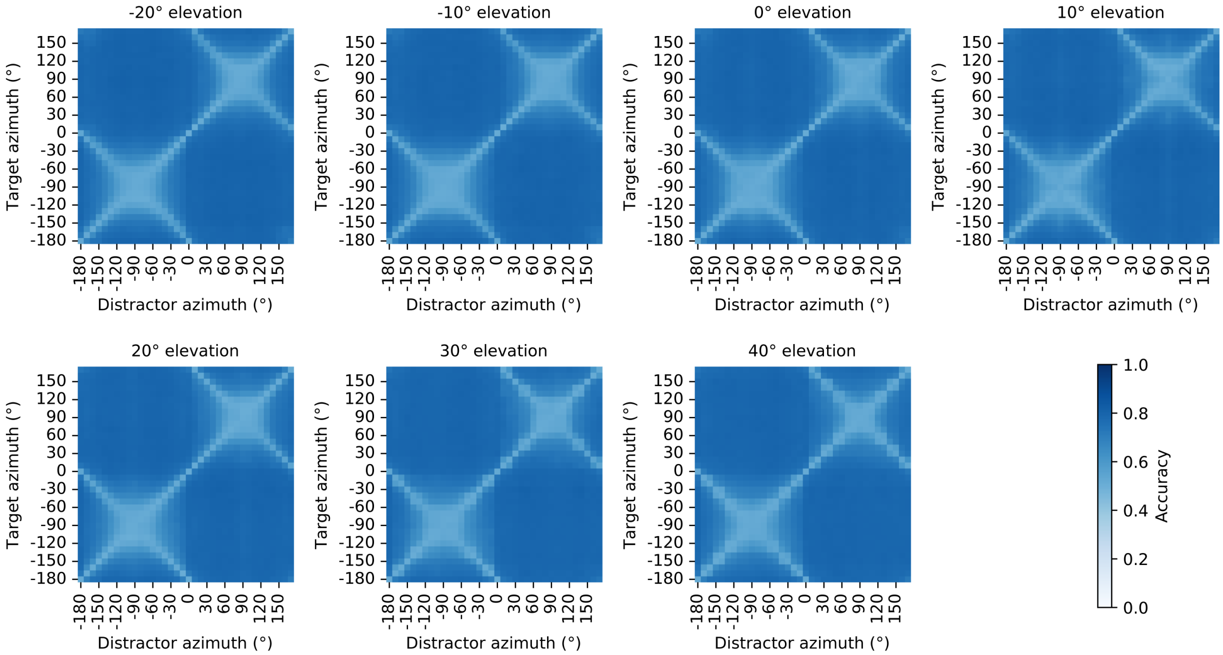

Supplementary Fig. 2 | Model word recognition at all target-distractor azimuth pairings. Word recognition performance for the main feature-gain model (arch_v00 in Supplementary Table 1) as a function of target and distractor positions in azimuth. Each panel plots results for a different elevation, with target azimuth on the vertical axis and distractor azimuth on the horizontal axis (targets and distractors were at the same elevation). The plots differ from those in Figure 4 in plotting results for the full 360 degrees of azimuthal positions (for completeness). Because there was little effect of whether a source was in the front or back hemisphere, the plots in Figure 4 averaged the two hemispheres. The front-back symmetry yields the "X" structures evident in the plots. The broadening of spatial acuity as the target azimuth moved peripherally (i.e. approaching $\pm 90°$ from $0°$) was apparent at all elevations.

bioRxiv preprint doi: https://doi.org/10.1101/2025.05.28.656682; this version posted May 28, 2025. The copyright holder for this preprint (which was not certified by peer review) is the author/funder, who has granted bioRxiv a license to display the preprint in perpetuity. It is made available under aCC-BY 4.0 International license.

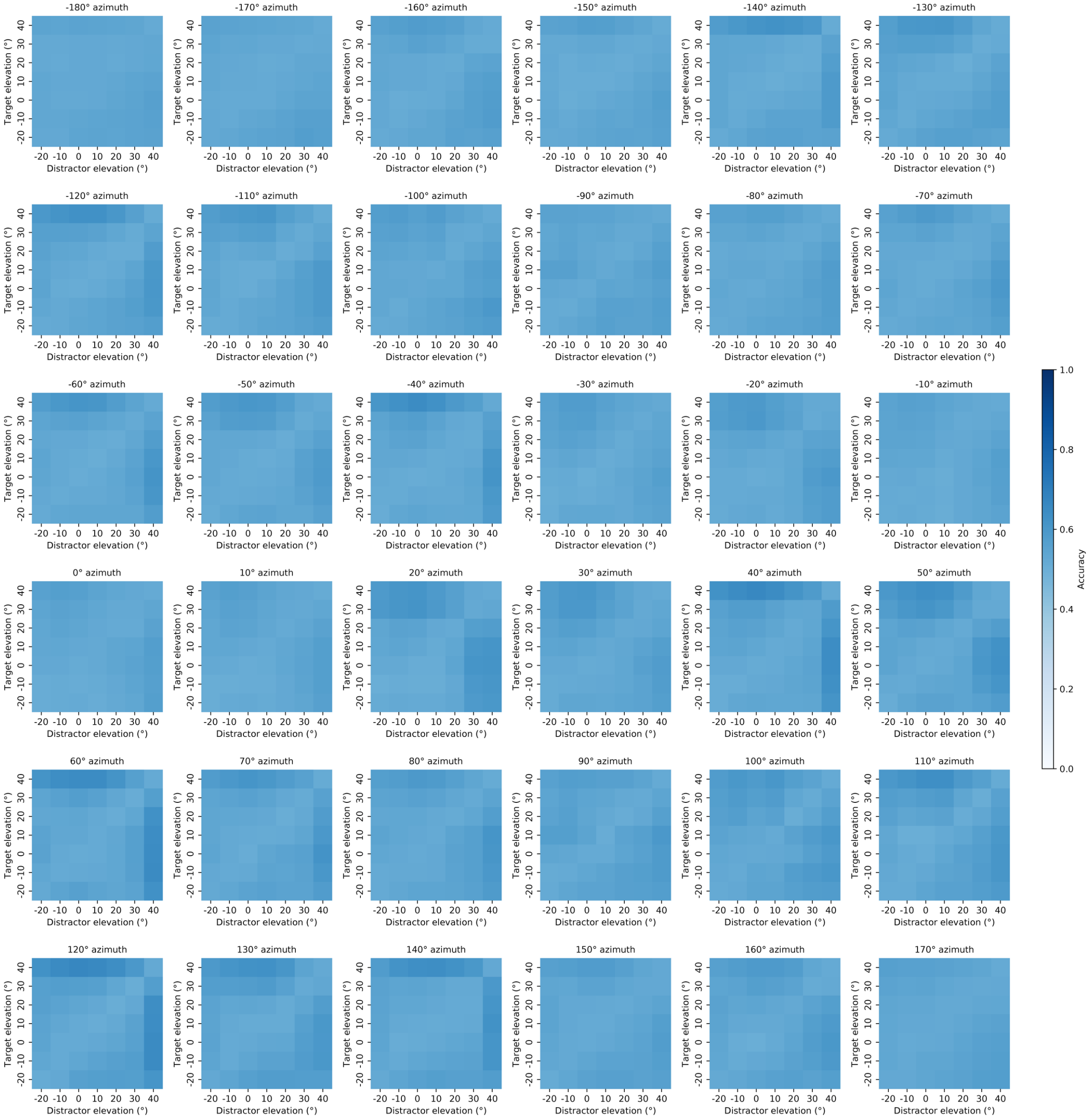

Supplementary Fig. 3 | Model word recognition at all target-distractor elevation pairings. Word recognition performance for the main feature gain model (arch_v00 in Supplementary Table 1) as a function of target and distractor positions in elevation. Each panel plots results for a different azimuth, with target elevation on the vertical axis and distractor elevation on the horizontal axis (targets and distractors were at the same azimuth). Target-distractor offset in elevation produced little benefit regardless of azimuthal position.

bioRxiv preprint doi: https://doi.org/10.1101/2025.05.28.656682; this version posted May 28, 2025. The copyright holder for this preprint (which was not certified by peer review) is the author/funder, who has granted bioRxiv a license to display the preprint in perpetuity. It is made available under aCC-BY 4.0 International license.

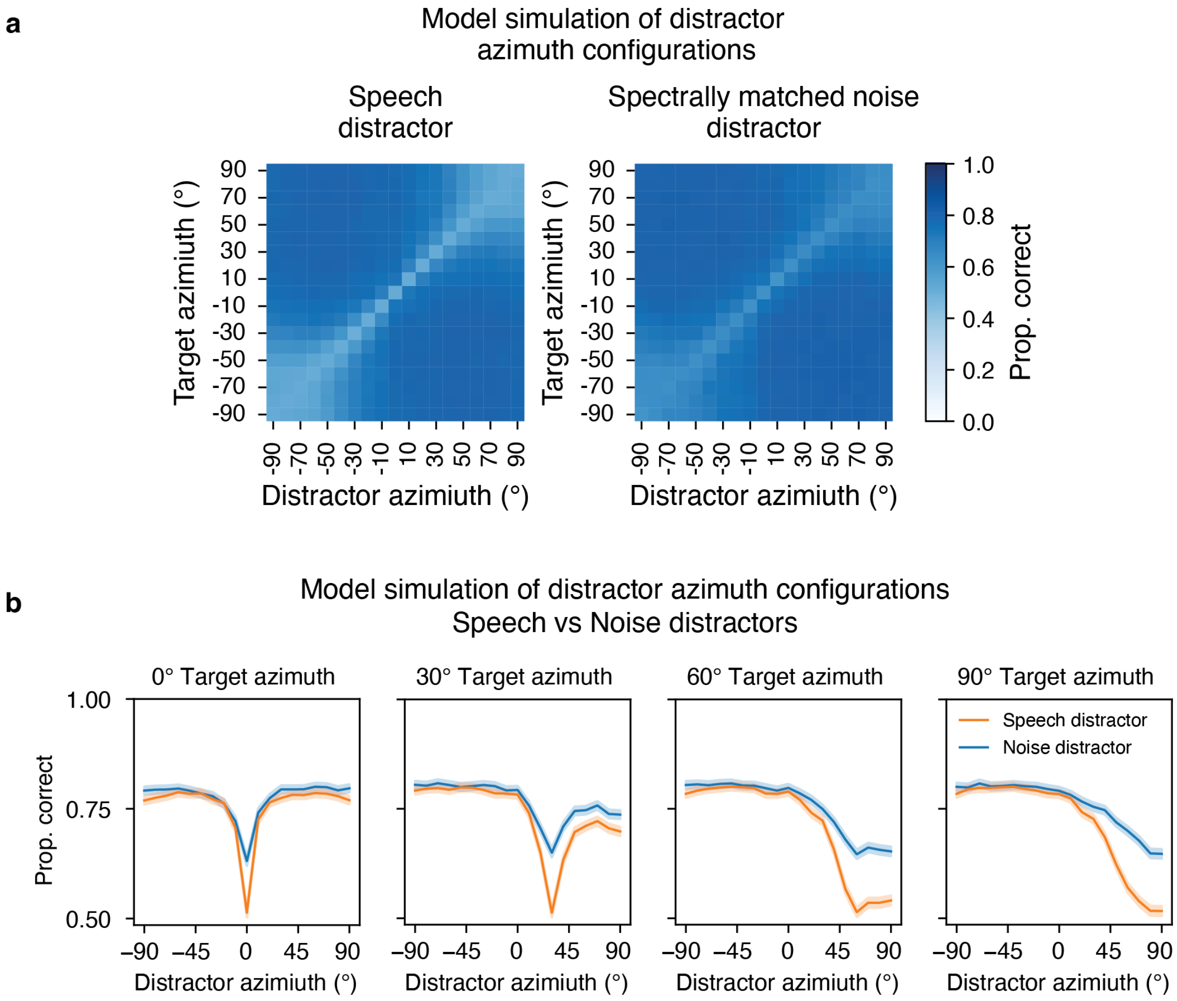

Supplementary Fig. 4 | Effect of target and distractor spatial position on model performance for speech and noise distractors. a. Word recognition performance for the main feature-gain model (arch_v00 in Supplementary Table 1) is plotted as a function of target and distractor positions in azimuth, for both single-talker distractors and speech-shaped noise distractors. As in Figure 4, results are averaged across front and back positions for ease of visualization. **b**. Data from (a) plotted as line graphs for four example target positions, to enable direct comparison of performance for speech and noise distractors. Shaded regions plot confidence intervals (95%) obtained via bootstrap. Performance with noise distractors varies much less with spatial position than performance with speech distractors, as in humans[113,114]. The presumptive explanation is that voice features alone are sufficient to select speech from noise, such that there is less benefit from spatial attention. There is nonetheless some spatial benefit on performance, consistent with prior results in human listeners[114]. This spatial dependence could be explained by a combination of changes in signal-to-noise ratio at the ear (which improves at the ear closer to the target as the distractor is moved away) and binaural effects related to masking[115,116]. However, the larger effect for speech distractors suggests a contribution of spatial attention in this setting. The similar spatial dependence for speech and noise distractors presumably reflects the spatial dependence of the underlying binaural cues, which also cause localization acuity to be worse in the periphery.

bioRxiv preprint doi: https://doi.org/10.1101/2025.05.28.656682; this version posted May 28, 2025. The copyright holder for this preprint (which was not certified by peer review) is the author/funder, who has granted bioRxiv a license to display the preprint in perpetuity. It is made available under aCC-BY 4.0 International license.

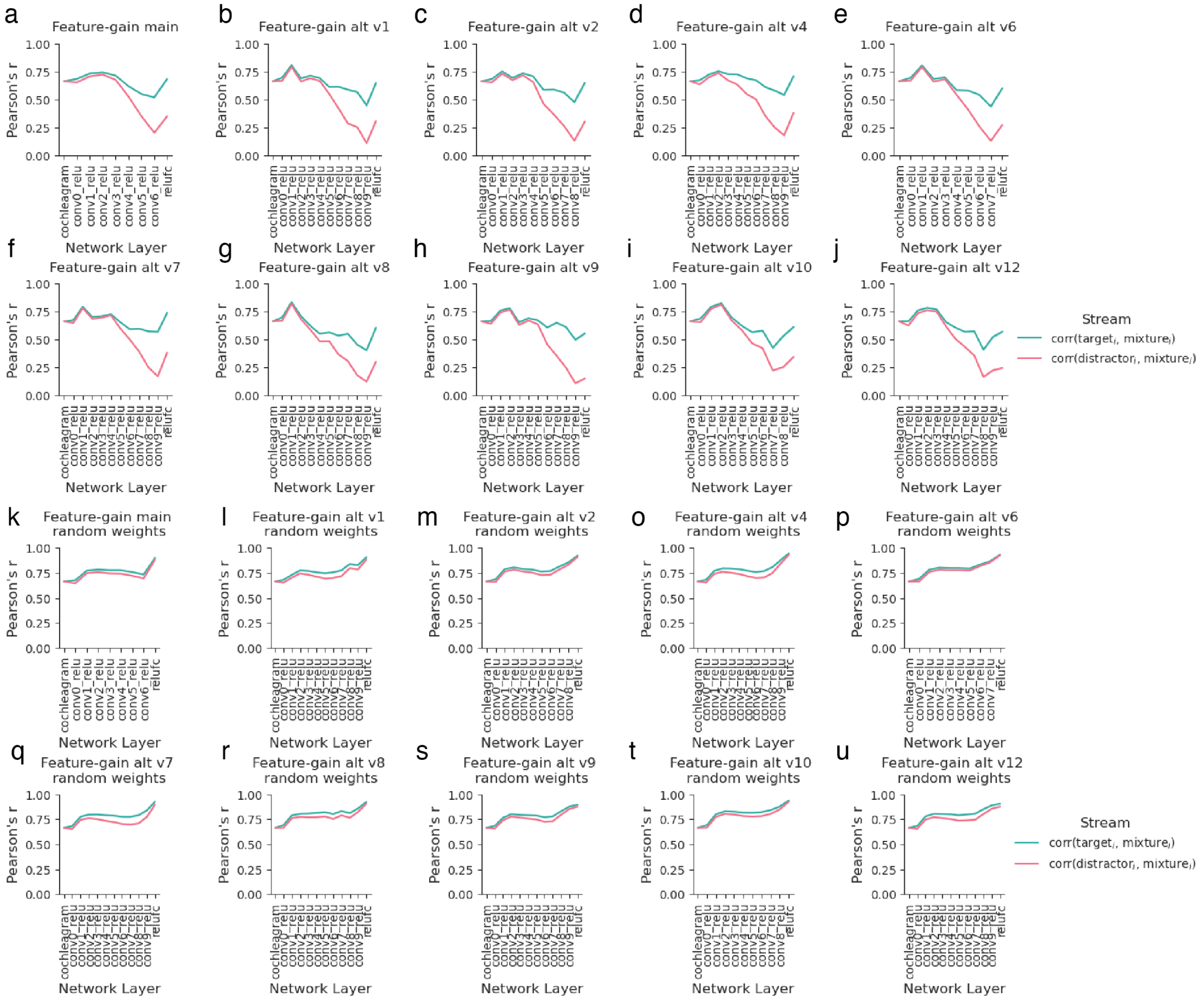

Supplementary Fig. 5 | Stage-of-selection analysis for each model architecture. Analysis of Fig. 5, plotted separately for each model architecture. All feature-gain architectures displayed fairly similar patterns of target enhancement at late model stages (panels a-j). Each architecture initialized with random weights did not show the same trend (panels k-u).

bioRxiv preprint doi: https://doi.org/10.1101/2025.05.28.656682; this version posted May 28, 2025. The copyright holder for this preprint (which was not certified by peer review) is the author/funder, who has granted bioRxiv a license to display the preprint in perpetuity. It is made available under aCC-BY 4.0 International license.

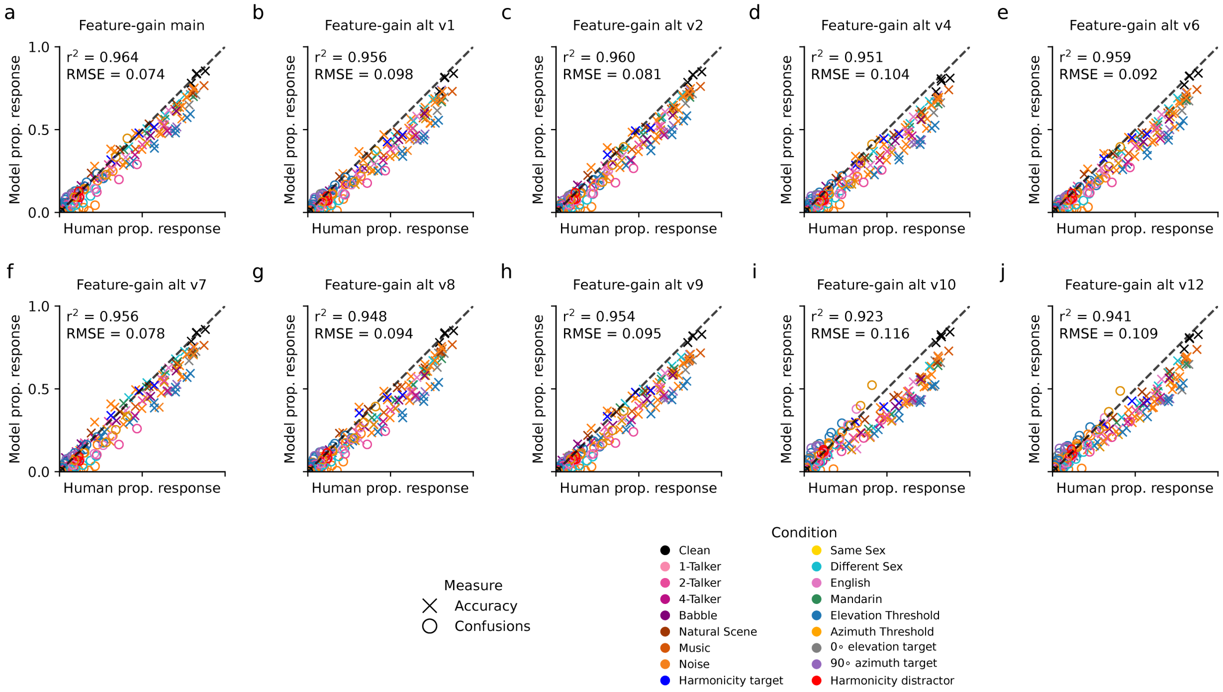

Supplementary Fig. 6 | Comparison of human and model performance for each model architecture
Scatter plots of model vs. human performance for each individual feature-gain architecture.

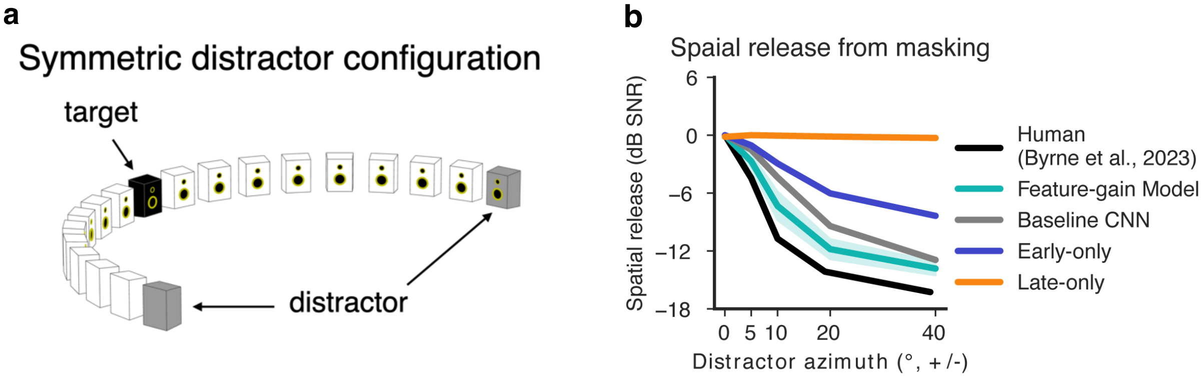

Supplementary Fig. 7 | Spatial release from masking for models lacking architectural constraints.
Performance of alternative model architectures (feature-gain, baseline, early-only, and late-only models) as a function of target-distractor separation in azimuth for symmetrically positioned distractors (Experiment 4). Models are compared to human data from Byrne et al. (2024). The y-axis plots spatial release from masking (the decrease in speech reception thresholds compared to the co-located distractor condition) at each tested azimuthal offset. Each of the alternative models displayed less human-like spatial release from masking than the feature-gain model. The late-only model showed no spatial masking release (orange), and the baseline (grey) and early-only model (purple) exhibited degraded masking release compared to the feature-gain model.

bioRxiv preprint doi: https://doi.org/10.1101/2025.05.28.656682; this version posted May 28, 2025. The copyright holder for this preprint (which was not certified by peer review) is the author/funder, who has granted bioRxiv a license to display the preprint in perpetuity. It is made available under aCC-BY 4.0 International license.

Architecture	arch_v00	arch_v01	arch_v02	arch_v04	arch_v06	arch_v07	arch_v08	arch_v09	arch_v10	arch_v12
**Operation**	Input [40,20000,2]	Input [40,20000,2]	Input [40,20000,2]	Input [40,20000,2]	Input [40,20000,2]	Input [40,20000,2]	Input [40,20000,2]	Input [40,20000,2]	Input [40,20000,2]	Input [40,20000,2]
1	Coch Lnorm	Coch Lnorm	Coch Lnorm	Coch Lnorm	Coch Lnorm	Coch Lnorm	Coch Lnorm	Coch Lnorm	Coch Lnorm	Coch Lnorm
2	Feature-gains 0	Feature-gains 0	Feature-gains 0	Feature-gains 0	Feature-gains 0	Feature-gains 0	Feature-gains 0	Feature-gains 0	Feature-gains 0	Feature-gains 0
3	Lnorm0	Lnorm0	Lnorm0	Lnorm0	Lnorm0	Lnorm0	Lnorm0	Lnorm0	Lnorm0	Lnorm0
4	conv0 [2,34, 32]	conv0 [1,59, 32]	conv0 [2,16, 32]	conv0 [3, 55, 64]	conv0 [1, 22, 32]	conv0 [2, 71, 32]	conv0 [1, 32, 32]	conv0 [2, 11, 32]	conv0 [1, 16, 32]	conv0 [3, 75, 32]
5	Relu0	Relu0	Relu0	Relu0	Relu0	Relu0	Relu0	Relu0	Relu0	Relu0
6	HannPool0 [2,4]	HannPool0 [2,1]	HannPool0 [2,5]	HannPool0 [2,3]	HannPool0 [2,3]	HannPool0 [2,5]	HannPool0 [2,6]	HannPool0 [2,2]	HannPool0 [2,3]	HannPool0 [2,2]
7	Feature-gains 1	Feature-gains 1	Feature-gains 1	Feature-gains 1	Feature-gains 1	Feature-gains 1	Feature-gains 1	Feature-gains 1	Feature-gains 1	Feature-gains 1
8	Lnorm1	Lnorm1	Lnorm1	Lnorm1	Lnorm1	Lnorm1	Lnorm1	Lnorm1	Lnorm1	Lnorm1
9	conv1 [2, 4, 64]	conv1 [2,72, 64]	conv1 [2, 74, 64]	conv1 [2,32,128]	conv1 [1, 34, 64]	conv1 [1, 33, 64]	conv1 [3, 47, 64]	conv1 [3, 60, 64]	conv1 [2, 79, 64]	conv1 [2, 44, 64]
10	Relu1	Relu1	Relu1	Relu1	Relu1	Relu1	Relu1	Relu1	Relu1	Relu1
11	HannPool1 [2,4]	HannPool1 [2,5]	HannPool1 [2,6]	HannPool1 [2,4]	HannPool1 [2,5]	HannPool1 [2,1]	HannPool1 [2,4]	HannPool1 [2,6]	HannPool1 [2,5]	HannPool1 [2,6]
12	Feature-gains 2	Feature-gains 2	Feature-gains 2	Feature-gains 2	Feature-gains 2	Feature-gains 2	Feature-gains 2	Feature-gains 2	Feature-gains 2	Feature-gains 2
13	Lnorm2	Lnorm2	Lnorm2	Lnorm2	Lnorm2	Lnorm2	Lnorm2	Lnorm2	Lnorm2	Lnorm2
14	conv2 [5,5, 256]	conv2 [4,3, 128]	conv2 [5, 4, 128]	conv2 [5, 3, 256]	conv2 [3, 5, 128]	conv2 [3, 6, 128]	conv2 [4, 4, 128]	conv2 [4, 5, 128]	conv2 [5, 4, 128]	conv2 [5, 4, 128]
15	Relu2	Relu2	Relu2	Relu2	Relu2	Relu2	Relu2	Relu2	Relu2	Relu2
16	HannPool2 [1,5]	HannPool2 [1,3]	HannPool2 [1,2]	HannPool2 [1,3]	HannPool2 [1,3]	HannPool2 [1,2]	HannPool2 [1,3]	HannPool2 [1,1]	HannPool2 [1,2]	HannPool2 [1,2]
17	Feature-gains 3	Feature-gains 3	Feature-gains 3	Feature-gains 3	Feature-gains 3	Feature-gains 3	Feature-gains 3	Feature-gains 3	Feature-gains 3	Feature-gains 3
18	Lnorm3	Lnorm3	Lnorm3	Lnorm3	Lnorm3	Lnorm3	Lnorm3	Lnorm3	Lnorm3	Lnorm3
19	conv3 [5,5, 512]	conv3 [6,3, 256]	conv3 [6, 5, 256]	conv3 [5, 4, 512]	conv3 [6, 4, 256]	conv3 [5, 3, 256]	conv3 [4, 6, 256]	conv3 [4, 4, 256]	conv3 [6, 6, 256]	conv3 [4, 6, 256]
20	Relu3	Relu3	Relu3	Relu3	Relu3	Relu3	Relu3	Relu3	Relu3	Relu3
21	HannPool3 [1,4]	HannPool3 [1,3]	HannPool3 [1,2]	HannPool3 [1,1]	HannPool3 [1,3]	HannPool3 [1,2]	HannPool3 [1,3]	HannPool3 [1,3]	HannPool3 [1,2]	HannPool3 [1,3]
22	Feature-gains 4	Feature-gains 4	Feature-gains 4	Feature-gains 4	Feature-gains 4	Feature-gains 4	Feature-gains 4	Feature-gains 4	Feature-gains 4	Feature-gains 4
23	Lnorm4	Lnorm4	Lnorm4	Lnorm4	Lnorm4	Lnorm4	Lnorm4	Lnorm4	Lnorm4	Lnorm4
24	conv4 [6 6, 512]	conv4 [4,4, 512]	conv4 [3, 3, 512]	conv4 [6, 6, 512]	conv4 [3, 4, 512]	conv4 [6, 6, 512]	conv4 [3, 6, 512]	conv4 [3, 4, 512]	conv4 [6, 4, 512]	conv4 [5, 6, 512]
25	Relu4	Relu4	Relu4	Relu4	Relu4	Relu4	Relu4	Relu4	Relu4	Relu4
26	HannPool4 [1,1]	HannPool4 [1,2]	HannPool4 [1,1]	HannPool4 [1,1]	HannPool4 [1,2]	HannPool4 [1,3]	HannPool4 [1,2]	HannPool4 [1,3]	HannPool4 [1,3]	HannPool4 [1,1]
27	Feature-gains 5	Feature-gains 5	Feature-gains 5	Feature-gains 5	Feature-gains 5	Feature-gains 5	Feature-gains 5	Feature-gains 5	Feature-gains 5	Feature-gains 5
28	Lnorm5	Lnorm5	Lnorm5	Lnorm5	Lnorm5	Lnorm5	Lnorm5	Lnorm5	Lnorm5	Lnorm5
29	conv5 [5,5, 512]	conv5 [5 3, 512]	conv5 [4, 5, 512]	conv5 [4, 3, 512]	conv5 [6, 4, 512]	conv5 [3, 5, 512]	conv5 [6, 5, 512]	conv5 [4, 3, 512]	conv5 [3, 5, 512]	conv5 [5, 5, 512]
30	Relu5	Relu5	Relu5	Relu5	Relu5	Relu5	Relu5	Relu5	Relu5	Relu5
31	HannPool5 [1,1]	HannPool5 [1,3]	HannPool5 [1,2]	HannPool5 [1,1]	HannPool5 [1,3]	HannPool5 [1,1]	HannPool5 [1,1]	HannPool5 [1,1]	HannPool5 [1,2]	HannPool5 [1,3]
32	Feature-gains 6	Feature-gains 6	Feature-gains 6	Feature-gains 6	Feature-gains 6	Feature-gains 6	Feature-gains 6	Feature-gains 6	Feature-gains 6	Feature-gains 6
33	Lnorm6	Lnorm6	Lnorm6	Lnorm6	Lnorm6	Lnorm6	Lnorm6	Lnorm6	Lnorm6	Lnorm6
34	conv6 [6 6, 512]	conv6 [5,4, 512]	conv6 [6, 6, 512]	conv6 [5, 3, 512]	conv6 [5, 6, 512]	conv6 [6, 3, 512]	conv6 [5, 4, 512]	conv6 [5, 3, 512]	conv6 [4, 4, 512]	conv6 [3, 3, 512]
35	Relu6	Relu6	Relu6	Relu6	Relu6	Relu6	Relu6	Relu6	Relu6	Relu6
36	HannPool6 [2,4]	HannPool6 [1,2]	HannPool6 [1,3]	HannPool6 [1,3]	HannPool6 [1,2]	HannPool6 [1,2]	HannPool6 [1,2]	HannPool6 [1,3]	HannPool6 [1,2]	HannPool6 [1,2]
37	Feature-gains fc	Feature-gains 7	Feature-gains 7	Feature-gains 7	Feature-gains 7	Feature-gains 7	Feature-gains 7	Feature-gains 7	Feature-gains 7	Feature-gains 7
38	flatten	Lnorm7	Lnorm7	Lnorm7	Lnorm7	Lnorm7	Lnorm7	Lnorm7	Lnorm7	Lnorm7
39	fc [512]	conv7 [3,5, 512]	conv7 [6, 6, 512]	conv7 [6, 4, 512]	conv7 [6, 4, 512]	conv7 [4, 4, 512]	conv7 [4, 4, 512]	conv7 [4, 6, 512]	conv7 [5, 3, 512]	conv7 [6, 6, 512]
40	Relufc	Relu7	Relu7	Relu7	Relu7	Relu7	Relu7	Relu7	Relu7	Relu7
41	dropout	HannPool7 [1,3]	HannPool7 [1,2]	HannPool7 [1,3]	HannPool7 [1,2]	HannPool7 [1,3]	HannPool7 [1,1]	HannPool7 [1,3]	HannPool7 [1,2]	HannPool7 [1,3]
42	fc [800]	Feature-gains 8	Feature-gains 8	Feature-gains 8	Feature-gains fc	Feature-gains 8	Feature-gains 8	Feature-gains 8	Feature-gains 8	Feature-gains 8
43		Lnorm8	Lnorm8	Lnorm8	flatten	Lnorm8	Lnorm8	Lnorm8	Lnorm8	Lnorm8
44		conv8 [6,4, 512]	conv8 [6, 4, 512]	conv8 [6, 6, 512]	fc [512]	conv8 [3, 4, 512]	conv8 [5, 5, 512]	conv8 [3, 3, 512]	conv8 [5, 6, 512]	conv8 [4, 4, 512]
45		Relu8	Relu8	Relu8	Relufc	Relu8	Relu8	Relu8	Relu8	Relu8
46		HannPool8 [1,1]	HannPool8 [1,2]	HannPool8 [1,3]	dropout	HannPool8 [1,3]	HannPool8 [1,2]	HannPool8 [1,2]	HannPool8 [1,2]	HannPool8 [1,2]
47		Feature-gains 9	Feature-gains fc	Feature-gains 9	fc [800]	Feature-gains 9	Feature-gains 9	Feature-gains 9	Feature-gains fc	Feature-gains 9
48		Lnorm9	flatten	Lnorm9		Lnorm9	Lnorm9	Lnorm9	flatten	Lnorm9
49		conv9 [6,3, 512]	fc [512]	conv9 [6, 3, 512]		conv9 [3, 3, 512]	conv9 [5, 4, 512]	conv9 [5, 6, 512]	fc [512]	conv9 [5, 5, 512]
50		Relu9	Relufc	Relu9		Relu9	Relu9	Relu9	Relufc	Relu9
51		HannPool9 [1,2]	dropout	HannPool9 [1,3]		HannPool9 [1,3]	HannPool9 [1,3]	HannPool9 [1,1]	dropout	HannPool9 [1,1]
52		Feature-gains fc	fc [800]	Feature-gains fc		Feature-gains fc	Feature-gains fc	Feature-gains fc	fc [800]	Feature-gains fc
53		flatten		flatten		flatten	flatten	flatten		flatten
54		fc [512]		fc [512]		fc [512]	fc [512]	fc [512]		fc [512]
55		Relufc		Relufc		Relufc	Relufc	Relufc		Relufc
56		dropout		dropout		dropout	dropout	dropout		dropout
57		fc [800]		fc [800]		fc [800]	fc [800]	fc [800]		fc [800]
58										

Supplementary Table 1 I Neural network architectures for feature-gain models. Grey bands indicate stages where feature-gain operations occurred. Legend:

- *Lnorm*: layer normalization operation
- conv $[h, w, k]$: convolutional layer with h = kernel height (frequency dimension), w = kernel width (time dimension), and k = number of kernels
- Relu: rectified linear unit activation function
- HannPool $[s_f, s_t]$: Hanning window weighted averaging pooling operation with stride s_f in the frequency dimension and stride s_t in the time dimension
- flatten: reshape operation to map a multidimensional tensor to a vector
- dropout: dropout regularization with 50% dropout rate
- fc $[N]$: fully-connected layer with N units
